# Tobacco-derived particulates and the periodontal axis: Distinct cytotoxic and stress-related mechanisms in human gingival fibroblasts

**DOI:** 10.1038/s41598-026-35317-8

**Published:** 2026-02-24

**Authors:** K. Kolci, E. Oz, S. Yildirim, R. Azevedo, H. S. Gungormek, A. Almeida, R. Reis

**Affiliations:** 1 Department of Toxicology, Faculty of Pharmacy, Acibadem Mehmet Ali Aydinlar University, Istanbul, Turkey; 2 Doctoral Program in Pharmaceutical Toxicology, Graduate School, Yeditepe University, Istanbul, Turkey; 3 Institute of Graduate Studies in Health Sciences, Istanbul University, Istanbul, Turkey; 4 Department of Analytical Chemistry, Faculty of Pharmacy, Karadeniz Technical University, Trabzon, Turkey; 5 LAQV/REQUIMTE, Department of Chemical Sciences, Faculty of Pharmacy, University of Porto, Porto, Portugal; 6 Department of Periodontology, Faculty of Dentistry, Marmara University, Istanbul, Turkey

**Keywords:** Tobacco products, Periodontal diseases, Total particulate matter, Oxidative stress, Inflammation, Autophagosome formation, Biochemistry, Cell biology, Diseases, Drug discovery, Medical research

## Abstract

Tobacco products vary widely in their chemical composition and potential harm, yet their impact on oral tissue remains insufficiently characterized. This study comparatively investigated the cytotoxic, oxidative, and inflammatory responses, along with apoptotic/necrotic cell death, autophagosome formation, and tissue remodeling capacity, in human gingival fibroblasts (hGFs) exposed to total particulate matter (TPM) derived from a conventional cigarette (TPM-c) and a heated tobacco product (TPM-h). TPMs were chemically characterized by inductively coupled plasma mass spectrometry (ICP-MS) for heavy metal content. TPM-c induced notable cytotoxicity, necrosis, and impaired wound healing compared to TPM-h, although both products compromised hGF viability and function. In addition, higher levels of Cadmium (Cd), Lead (Pb), and Zinc (Zn) were detected in TPM-c. Triggered vascular endothelial growth factor-A (VEGF-A) upregulation as a defensive reaction to cellular stress was observed in hGFs via TPM-c, while TPM-h reduced autophagic response via Microtubule-associated protein 1 A/1 B-light chain 3-phosphatidyl ethanolamine conjugate/LC3-II (LC3β) expression. Both TPMs elevated interleukin-6 (IL-6) release, notably at intermediate and high doses. In summary, TPM-c demonstrated a greater capacity than TPM-h to induce cytotoxicity, oxidative and inflammatory damage, and disrupted tissue remodeling. Nonetheless, TPM-h was not devoid of toxicity, eliciting pro-inflammatory/ angiogenic responses concentration-dependently. These findings highlight the necessity of further investigation into the long-term effects of emerging tobacco products on periodontal disease progression and development.

**Supplementary Information:**

The online version contains supplementary material available at 10.1038/s41598-026-35317-8.

## Introduction

Tobacco smoking continues to be one of the leading causes of preventable morbidity and mortality worldwide, accounting for more than seven million deaths each year, including over one million passive smokers^1^. In Türkiye, smoking prevalence is among the highest within the top three Organization for Economic Co-operation and Development (OECD) countries, with 28% of the population smoking daily and 3.4% smoking occasionally^2^. The majority of daily smokers are men aged 25–44, and initiation is often linked to social and personal factors such as peer influence, curiosity, or family-related problems. A recent regional analysis ranked Türkiye as the third country in the Middle East and North Africa region with a smoking prevalence of 30.9%, surpassing the regional average of 19.6%^3^. Such statistics highlight tobacco use as a major public health concern in the country. Moreover, the toxicological burden of smoking arises from exposure to aerosols containing more than 7000 chemical constituents, including nicotine, carbon monoxide (CO), aldehydes, oxidants, heavy metals, and TPM. These compounds play crucial roles in the onset and progression of systemic diseases such as cancer, cardiovascular disorders, chronic obstructive pulmonary disease (COPD), and liver disease^4^. To mitigate these risks, the tobacco industry has introduced and aggressively marketed alternative products, such as electronic cigarettes (e-cigarettes) and heated tobacco products (HTPs), under the claim of “reduced risk.” E -cigarettes deliver aerosols from a water-based solution of nicotine, propylene glycol, glycerol, and flavoring agents without containing tobacco^5,6^. HTPs, in contrast, heat tobacco at ~ 350 °C, lower than the ~ 600 °C combustion temperature of traditional cigarettes, thereby reducing but not eliminating the formation of carcinogens such as polycyclic aromatic hydrocarbons, benzene, arsenic (As), and tobacco-specific nitrosamines (TSNAs)^5^. Consequently, both conventional cigarettes and HTPs display distinct chemical and toxicological profiles that can induce different biological responses^7,8^. As an important first contact target organ, the oral cavity, and particularly the gingiva, are critical sites of direct exposure to tobacco aerosols. Cigarette smoke (CS) is an established risk factor for oral mucosal and periodontal diseases, including gingivitis, periodontitis, tooth discoloration, and oral cancer^9–12^. Among the aerosol components, TPM exhibits a high deposition potential in gingival and dental tissues and contains pigmented compounds capable of inducing tooth discoloration and long-term aesthetic alterations^13^. Moreover, TPM maintains prolonged contact with the oral mucosa due to its particulate nature, thereby increasing the potential of cellular uptake and local toxicity. Evidence suggests that smoking adversely affects epithelial and connective tissue integrity, delays wound healing, and exacerbates periodontal tissue destruction. Previous clinical data demonstrated that smokers have higher prevalence of periodontitis, greater attachment loss, more tooth loss, increased calculus formation, and reduced treatment response compared to non-smokers^14^. Importantly, these effects are cumulative with the duration of smoking but are partially reversible upon cessation. Moreover, at the molecular level, smoking-induced periodontal pathology is strongly associated with oxidative stress and inflammation. Elevated levels of pro-inflammatory cytokines such as interleukin-1β (IL-1β), IL-6, and tumor necrosis factor-α (TNF-α), together with oxidative stress biomarkers, have been consistently detected in gingival crevicular fluid and saliva of smokers^15^. Clinical studies also reported alterations in markers such as prostaglandin E2, matrix metalloproteinase-9 (MMP-9), and VEGF, underscoring a link between tobacco exposure and impaired tissue remodeling^16^. Interestingly, some findings suggest paradoxically lower bleeding indices in smokers despite heightened inflammatory mediator levels, possibly reflecting suppressed inflammatory cell infiltration^10,17^. These mechanistic insights strengthen the role of tobacco aerosols in periodontal tissue destruction. Despite these evidences, most existing studies have focused on conventional cigarettes or, more recently, e-cigarettes, while data on HTPs remain limited. Clinical findings indicate that HTP use may induce gingival epithelial changes and keratinocyte differentiation, potentially contributing to oral mucosal diseases such as leukoplakia or lichen planus^18^. However, systematic comparisons between conventional cigarettes and HTPs, particularly regarding TPM effects on periodontal-relevant cells, are largely lacking.

Therefore, this study aimed to comparatively evaluate the cytotoxic, oxidative, inflammatory, autophagic, and tissue-remodeling effects of TPM isolated from conventional cigarettes (TPM-c) and HTPs (TPM-h) in primary hGFs. By integrating endpoints such as cell death pathways, wound-healing capacity, cytokine release, VEGF, and MMP expression, this study seeks to provide novel mechanistic insights into how distinct tobacco-derived particulates interact with gingival tissues. To our knowledge, this is the first in vitro study systematically addressing the toxicological profiles of TPMs on gingival fibroblasts, thereby filling an important knowledge gap and contributing to the understanding of periodontal risks associated with emerging tobacco products.

## Results

### Nicotine level and heavy metal spectrum of TPMs

TPM-h (36 mg/mL) and TPM-c (24 mg/mL) were extracted with sterile dimethyl sulfoxide (DMSO, PanReac AppliChem, Darmstadt, Germany) for 24 h at 37 °C to contain an equal level of nicotine. Collected Whatman papers after TPM extractions were also given in Supplementary Fig. S1, and the weighted TPMs accumulated on Whatman paper were given in Table S1. The nicotine levels of the samples were determined via a nicotine standard calibration curve (Supplementary Fig. S2). According to our findings, the nicotine concentration in TPM-c samples was found to be 759.43 ± 2.80 µg/mL, whereas the nicotine concentration of TPM-h was reported as approximately 1.5-fold lower in our unpublished study^36^. These results indicated that TPM-c contained significantly higher nicotine levels compared to TPM-h isolated from one cigarette/ stick. Therefore, TPM extracts were adjusted to a 1:1.5 (v/v) ratio to ensure equal nicotine concentrations across all tested samples for subsequent analyses. According to our findings, most notably, Rubidium (Rb) concentration was 0.08 µg/g in TPM-h, whereas it was 1.4 ± 0.11 µg/g in TPM-c (17-fold higher). Lithium (Li) levels were detected at 0.07 µg/g in TPM-h, whereas TPM-c showed 0.20 ± 0.03 µg/g. Similarly, substantial increases were found in Zn, Rb, and Cd levels in TPM-c samples. In contrast, Mercury (Hg) and Thallium (Tl) concentrations were found to be low (~ 0.03 µg/g) in both extracts. Limit of detection (LoD) values and calculation formulas (Supplementary Formula S2) used for statistical comparisons are presented as mean ± SD (standard deviation) in Table 1*(n = 2)*. Overall, TPM-c contained higher levels of Cd, Tl, Pb, Li, Zn, and Rb, whereas Copper (Cu) measurements showed variability due to its high susceptibility to contamination and degradation during analysis. Overall, TPM-c showed higher levels of Cd, Tl, Pb, Li, Zn, and Rb, whereas Cu measurements showed variability due to its high susceptibility to contamination and degradation during analysis. Most notably, Rb concentration was 0.08 µg/g in TPM-h and 1.40 ± 0.11 µg/g in TPM-c – approximately 17-fold higher. Similarly, Li levels were determined at 0.07 µg/g in TPM-h and 0.20 ± 0.03 µg/g in TPM-c. Significantly higher levels were also found for Zn and Cd in TPM-c samples. Hg and Tl concentrations were found to be very low (~ 0.03 µg/g) in both TPMs.

**Table 1:** Quantification of heavy metals in TPM samples.

Heavy metal (µg/g)	TPM-h	TPM-c	*p* values	*n*
^85^Rb	0.08 ± 0.00	1.4 ± 0.11**	p^**^ *< 0.0001*	*2*
^66^Zn	1.05 ± 0.24	3.93 ± 0.45**	p^**^ *< 0.0001*	*2*
^7^Li	0.07 ± 0.00	0.2 ± 0.03**	p^**^ *< 0.0001*	*2*
^205^Tl	0.0 ± 0.00	0.08 ± 0.02**	p^**^ *< 0.0001*	*2*
^111^Cd	0.0 ± 0.00	3.54 ± 0.94**	p^**^ *< 0.0001*	*2*
^75^As	0.05 ± 0.00	0.08 ± 0.03*	p^*^ *< 0.05*	*2*
^202^Hg	0.03 ± 0.02	0.03 ± 0.01	ns	*2*
^208^Pb	0.03 ± 0.01	0.92 ± 0.07**	p^**^ *< 0.0001*	*2*
^65^Cu	0.52 ± 0.00	1.01 ± 0.85	ns	*2*

TPM-c: Total particulate matter derived from a conventional cigarette; TPM-h: Total particulate matter derived from a heated tobacco product; Rb: Rubidium; Zn: Zinc; Li: Lithium; Tl: Thallium; Cd: Cadmium; As: Arsenic; Hg: Mercury; Pb: Lead; Cu: Copper. Statistical significance between TPM-h vs. TPM-c as p* < 0.05; p** < 0.0001; ns: No significance. Data are expressed as mean ± SD of two independent experiments.

### Cytotoxicity of TPM-h and TPM-c in hGF cells

**Fig. 1:**
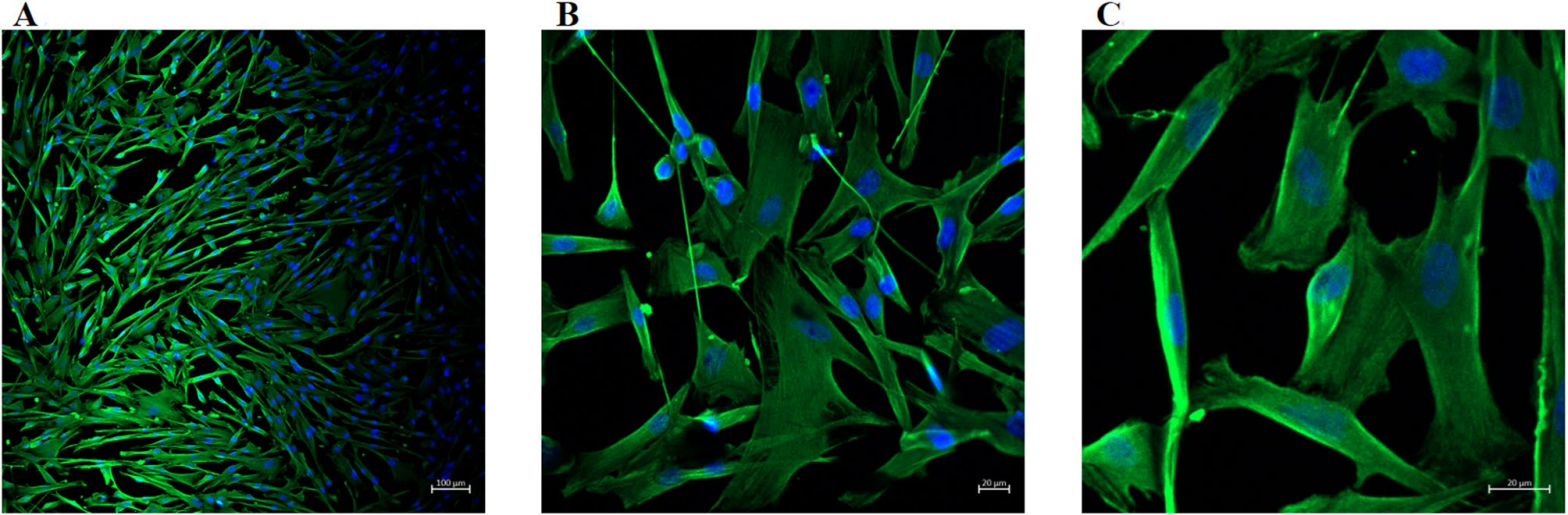
Confocal microscopic images of primary hGF cells stained with vimentin and DAPI. A: Confocal microscopic images of fibroblast cells stained with vimentin and DAPI taken at 5x confocal microscopy, B: Confocal microscopic images of fibroblast cells stained with vimentin and DAPI taken at 20x confocal microscopy, C: Confocal microscopic images of fibroblast cells stained with vimentin and DAPI taken at 40x. hGF: Human gingival fibroblasts; DAPI: 4’, 6-diamidino-2-phenylindole.

hGFs were isolated from the gingival tissue of a healthy donor, with their day-by-day generation illustrated in Supplementary Fig. S3, and the images of passaged hGFs up to day 6 were given in Supplementary Fig. S4. Prior to the cytotoxicity assay, the cells were characterized by confocal imaging after immunofluorescence staining with vimentin and 4’, 6-diamidino-2-phenylindole (DAPI, Mounting Medium with DAPI, Ibidi, #50011, in 1:1000 DPBS). The results revealed a homogeneous fibroblast cytoskeletal profile positive for vimentin, with nuclei counterstained by DAPI, as shown in Fig. 1.

The cytotoxic profiles of TPMs in hGF cells were evaluated by the MTT [3-(4,5-dimethylthiazol-2-yl)-2,5-diphenyltetrazolium bromide] assay, described in our previous study^19^. The doses were prepared at the maximum experimentally achievable concentration, and then cytotoxicity rates in hGF cells were determined using the MTT assay, which we optimized in our previous studies. Accordingly, the toxicity and wound healing capacity of TPM-h and TPM-c were evaluated using hGF cells. Briefly, the cells were seeded in 96-well microplates and incubated for 24 h (37 °C, 5% CO_2_). They were then exposed to doses of TPM-h (2.81–90 µg/mL) and TPM-c (1.8–60 µg/mL). Cytotoxicity was determined using the MTT assay, which measures mitochondrial function. After exposure, supernatants were removed, and 0.5 mg/mL MTT solution was added and incubated at 37 °C for 2 h. After incubation, the supernatants were discarded, and formazan crystals were dissolved in 100 µL of isopropanol. Absorbance values ​​were measured at 570 nm using a spectrophotometer (CLARIOstar, BMG LAbtech, Offenburg, Germany). Cellular viability was determined using the formula given in equation 1.

**Equation 1**: Relative cell viability.

$${\text{Relative Cell Viability }}\left( \% \right)\,=\,{\mathrm{1}}00{\text{ }} \times {\text{ O}}{{\mathrm{D}}_{{\mathrm{57}}0}}_{{{\mathrm{sample}}}}/{\mathrm{O}}{{\mathrm{D}}_{{\mathrm{57}}0{\mathrm{NC}}}}$$

OD_570 sample_: Average of the measured optical density of the sample. OD_570NC_: Average of the measured optical density of the negative control.

**Fig. 2:**
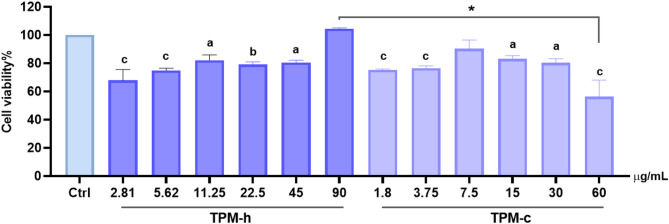
Relative cell viability of hGF cells exposed to TPM-h and TPM-c. Ctrl: Control group without any treatment; TPM-c: Total particulate matter derived from a conventional cigarette; TPM-h: Total particulate matter derived from a heated tobacco product. Statistical significance between groups: Ctrl vs. groups *p*^a^ < 0.005; *p*^b^ < 0.0005; *p*^c^ < 0.0001; TPM-h (90 µg/mL) vs. TPM-c (60 µg/mL) p^*^ < 0.0001. Data are expressed as mean ± SD of three independent experiments.

As shown in Fig. 2, cell viability decreased in a dose-dependent manner following exposure to both TPMs, which was found to be statistically significant. The highest experimentally achievable doses were 90 µg/mL for TPM-h and 60 µg/mL for TPM-c. This is because the lower TPM concentration must be dissolved with a minimum amount of solvent (DMSO) to reach the maximum concentration. The highest experimental concentrations were compared, and a significant difference in cell viability was detected; cell viability was decreased in TPM-c (*p* < 0.0001). Based on our findings, the doses used for further analyses were determined based on the half inhibiting concentration (IC_50_) values, for TPM-c as 60 µg/mL (56.35%±11.7 (mean ± SD)), whereas for TPM-h, the IC_50_ value exceeded the maximum experimentally testable concentration. Therefore, the dose selection for TPM-h was based on the concentration corresponding to the nicotine-equivalent level of TPM-c’s IC_50_ (60 µg/mL), and the doses were determined as 22.5, 45, and 90 µg/mL for TPM-h, and 15, 30, and 60 µg/mL for TPM-c. Notably, at the highest tested concentrations, TPM-c exerted a notably higher cytotoxic response in hGFs compared to TPM-h (*p* < 0.0001) (Fig. 2).

### TPM-mediated apoptotic and necrotic cell death

**Fig. 3:**
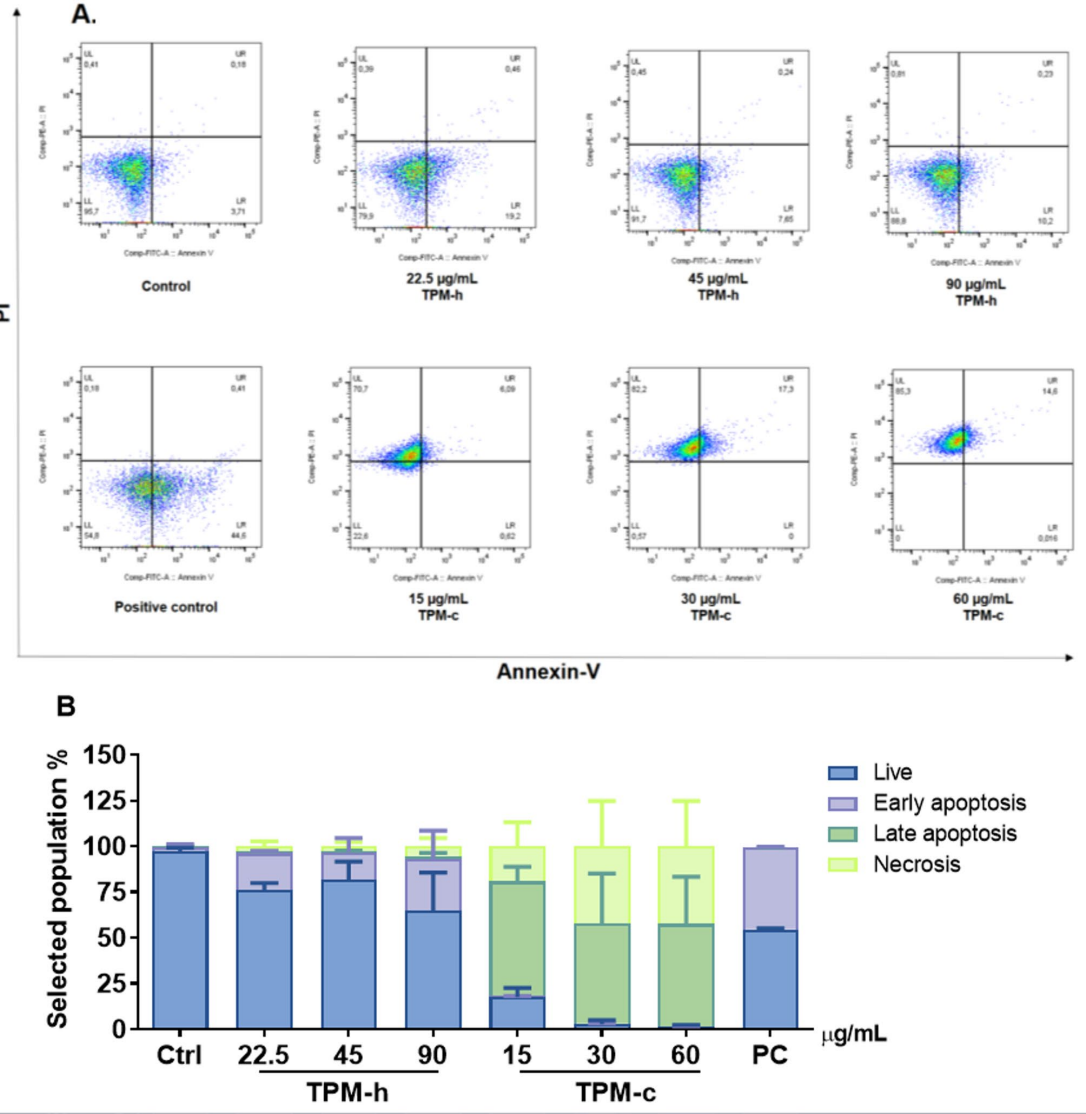
TPM-h and TPM-c induced apoptotic and necrotic cell death in hGF cells. hGF cells were exposed to TPM-h and TPM-c concentrations for 24 h and then stained with the Annexin V-PI kit. A: Representative histograms of apoptotic and necrotic cell death in hGFs exposed to TPMs. B: Total apoptosis rates of hGF cells exposed to TPMs. Ctrl: Control group without any treatment; TPM-c: Total particulate matter derived from a conventional cigarette; TPM-h: Total particulate matter derived from a heated tobacco product; PC: Positive control for early apoptosis (5% DMSO, v/v). Data are expressed as mean ± SD of three independent experiments.

After 24 h of exposure to both TPMs, flow cytometry analysis revealed that the cell death rate in the control group was 4.3%. In the TPM-h group, cell death rates were 20.05% (22.5 µg/mL), 8.34% (45 µg/mL), and 11.24% (90 µg/mL). In contrast, TPM-c exposure led to a marked increase in cell death, reaching 77.41% (15 µg/mL), 99.5% (30 µg/mL), and 99.92% (60 µg/mL). Notably, at higher doses, both groups showed increased total apoptotic cell percentages (Fig. 3). Similar to the positive control (PC: 5% DMSO, v/v), both TPMs induced cell death processes. An increase in total apoptotic cell rates was observed in both treatment groups, with a more pronounced effect at higher concentrations. Comparable to the PC, both TPMs treatments activated cell death, particularly via the apoptotic pathway. Notably, TPM-h predominantly induced early apoptosis, whereas TPM-c led to late apoptosis and even necrotic cell death. Representative flow cytometry histograms of hGF populations are presented in Fig. 3A, while quantitative cell death rates are shown in Fig. 3B.

### Autophagosome formation

**Fig. 4:**
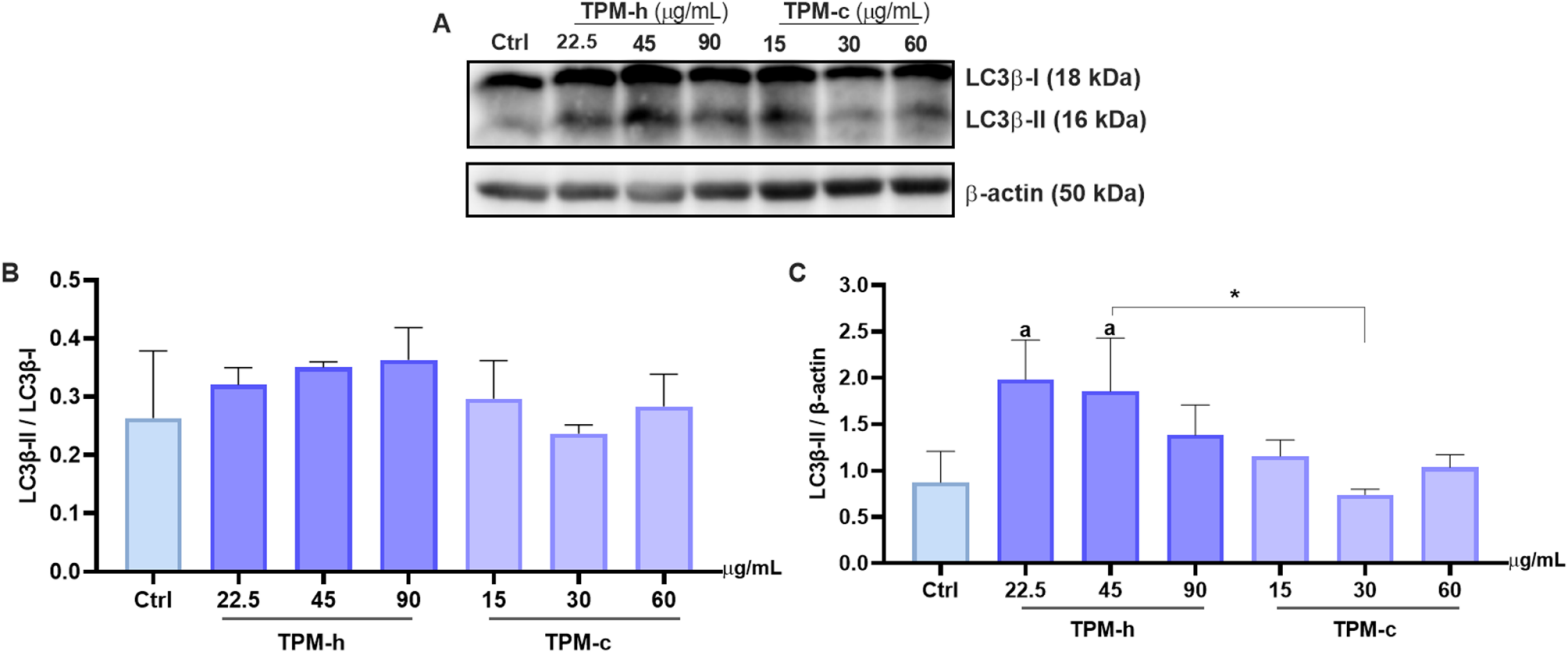
LC3β expression in hGF cells exposed to TPM-h and TPM-c. **A: **Band images of LC3β expressions and β-actin, **B:** LC3β-II/ LC3β-I, and **C:** LC3β-II/ β-actin. Ctrl: Control group without any treatment; TPM-c: Total particulate matter derived from a conventional cigarette; TPM-h: Total particulate matter derived from a heated tobacco product. Statistical significance between groups: (C): Ctrl vs. groups *p* < 0.05; TPM-h (45 µg/mL) vs. TPM-c (30 µg/mL) p* <0.05. Data are expressed as mean ± SD of three independent experiments.

The protein expression of microtubule-associated protein 1 A/1B-light chain 3-3-phosphatidyl-ethanolamine conjugate/LC3-II (LC3β), as an important marker for autophagosome formation, were up-regulated significantly in TPM-h (45 µg/mL) and TPM-c (30 µg/mL) as shown in Fig. 4A-C (*p < 0.05*). Prominently, the difference was observed at LC3-II/β-actin ratios between these groups (Fig. 4C). Our findings indicated an increased autophagic activity and a potential cellular stress response. Moreover, dose-dependent increase was observed in the TPM-h exposed hGF cells compared to the control group, but not in TPM-c exposure (Fig. 4).

### TPM-mediated oxidative damage and inflammatory response

**Fig. 5:**
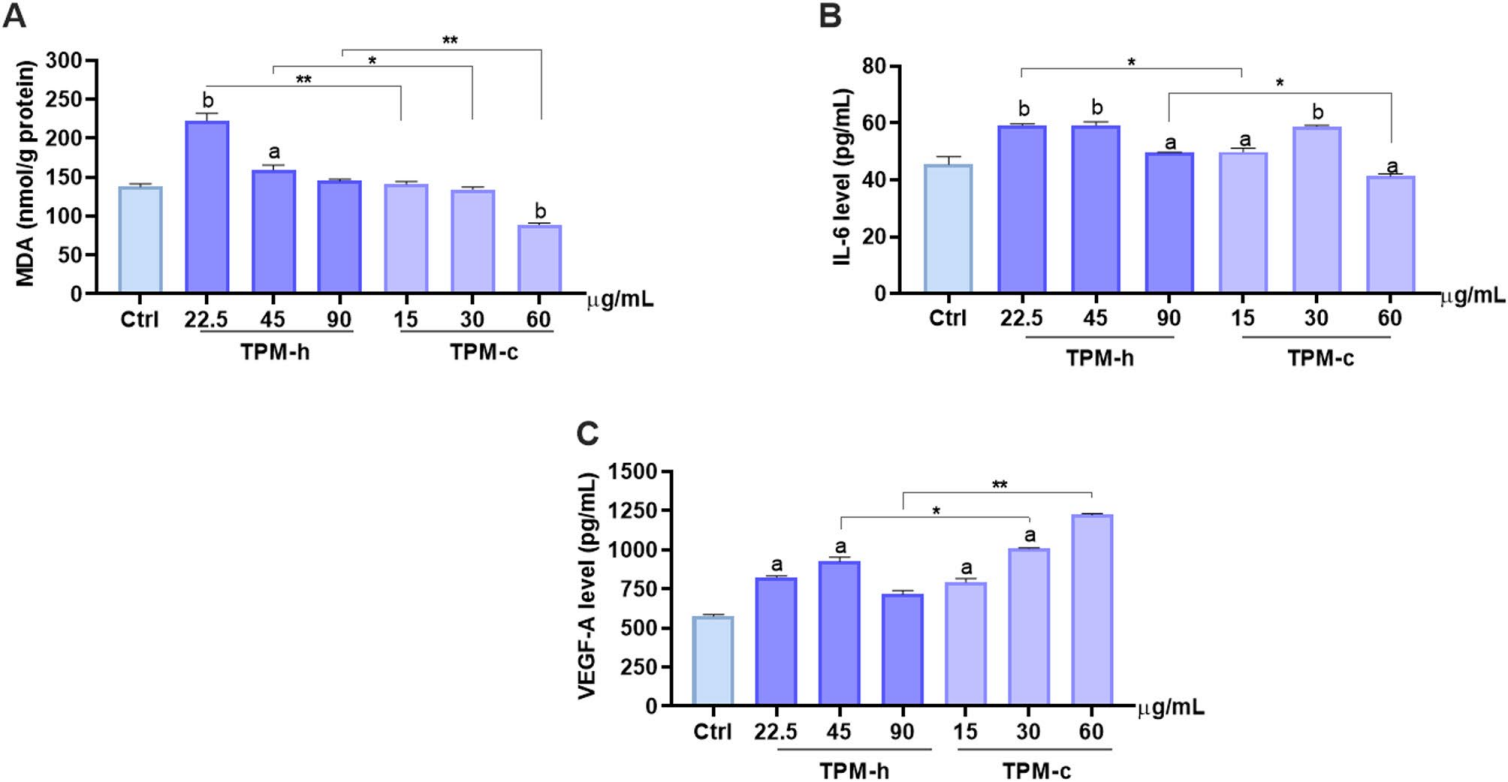
TPM-mediated oxidative damage and inflammatory response. **A**: Lipid peroxidation level in hGF cells after TPMs exposure. hGF cells were exposed to TPMs concentrations for 24 h and the lipid peroxidation level was measured. Ctrl: Control group without any treatment; TPM-c: Total particulate matter derived from a conventional cigarette; TPM-h: Total particulate matter derived from a heated tobacco product. Statistical significance between groups: Ctrl vs. groups *p*^a^ <0.001; *p*^b^ <0.0001; TPM-h (22.5 µg/mL) vs. TPM-c (15 µg/mL) p^**^ <0.0001; TPM-h (45 µg/mL) vs. TPM-c (30 µg/mL) p^*^ <0.005; TPM-h (90 µg/mL) vs. TPM-c (60 µg/mL) p^**^ < 0.0001; **B**: IL-6 level. Statistical significance between groups: control vs. groups *p*^a^ <0.05; *p*^b^ <0.0001; TPM-h (22.5 µg/mL) vs. TPM-c (15 µg/mL) p^*^ <0.0001; TPM-h (90 µg/mL) vs. TPM-c (60 µg/mL) p^*^ <0.0001; **C**: VEGF-A level, Statistical significance between groups: Ctrl vs. groups *p*^a^ <0.0001; TPM-h (45 µg /mL) vs. TPM-c (30 µg /mL) p^*^ < 0.001; TPM-h (90 µg /mL) vs. TPM-c (60 µg /mL) p^*^^*^ <0.0001. Data are expressed as mean ± SD of three independent experiments.

Glutathione (GSH) levels were evaluated in hGF cells after TPMs exposure, and no statistically significant difference was observed between the exposed groups. On the other hand, low and intermediate concentrations of TPM-h (22.5 and 45 µg/mL) led to significantly increased lipid peroxidation through malondialdehyde (MDA) level (*p*^*a*^ *< 0.001; p*^*b*^ *< 0.0001*), whereas equal nicotine-containing concentrations of TPM-c did not exhibit a similar dose-dependent path, possibly due to relative hGF cell viability at the final endpoint time (24 h). In all nicotine-equivalent concentration comparisons (TPM-h vs. TPM-c), higher signals were detected in the TPM-h group, indicating that TPM-h caused a pronounced oxidative damage through intracellular GSH release. Moreover, MDA levels, similar to the GSH assay, higher signals were detected in TPM-h (Fig. 5A).

The increase in IL-6 levels in the TPMs compared to the control group is given in Fig. 5B. Accordingly, an increase was observed in IL-6 levels compared to the control group, but these increases were independent of the concentration and were not at a level that would create a significant inflammatory response. In equivalent-nicotine level comparisons, an increase in IL-6 levels was detected in the TPM-h groups compared to the control group. A similar increase was observed in TPM-c administration, but the response was determined to be limited. These results showed that TPMs may slightly increase the inflammatory response.

In Fig. 5C, VEGF-A expression was evaluated after TPMs exposure, and a significant increase was observed in both groups (*p*^*a*^ *< 0.0001*). Especially in intermediate and high concentrations, TPM-c administration caused more VEGF production compared to TPM-h (p^*^< 0.001; p^**^ < 0.0001). A dose-dependent increase in VEGF-A level was observed in TPM-c. No significant difference was observed in MMP-9 levels after the TPMs exposure between the control and the TPM applied groups. MMP-9 levels were at similar levels in all groups, and it was determined that the applications did not significantly affect MMP-9 expression. These results show that TPM derivatives did not cause a significant change in MMP-9 levels in hGF cells within 24 h of exposure.

### Delay in wound healing and tissue-remodeling

**Fig. 6:**
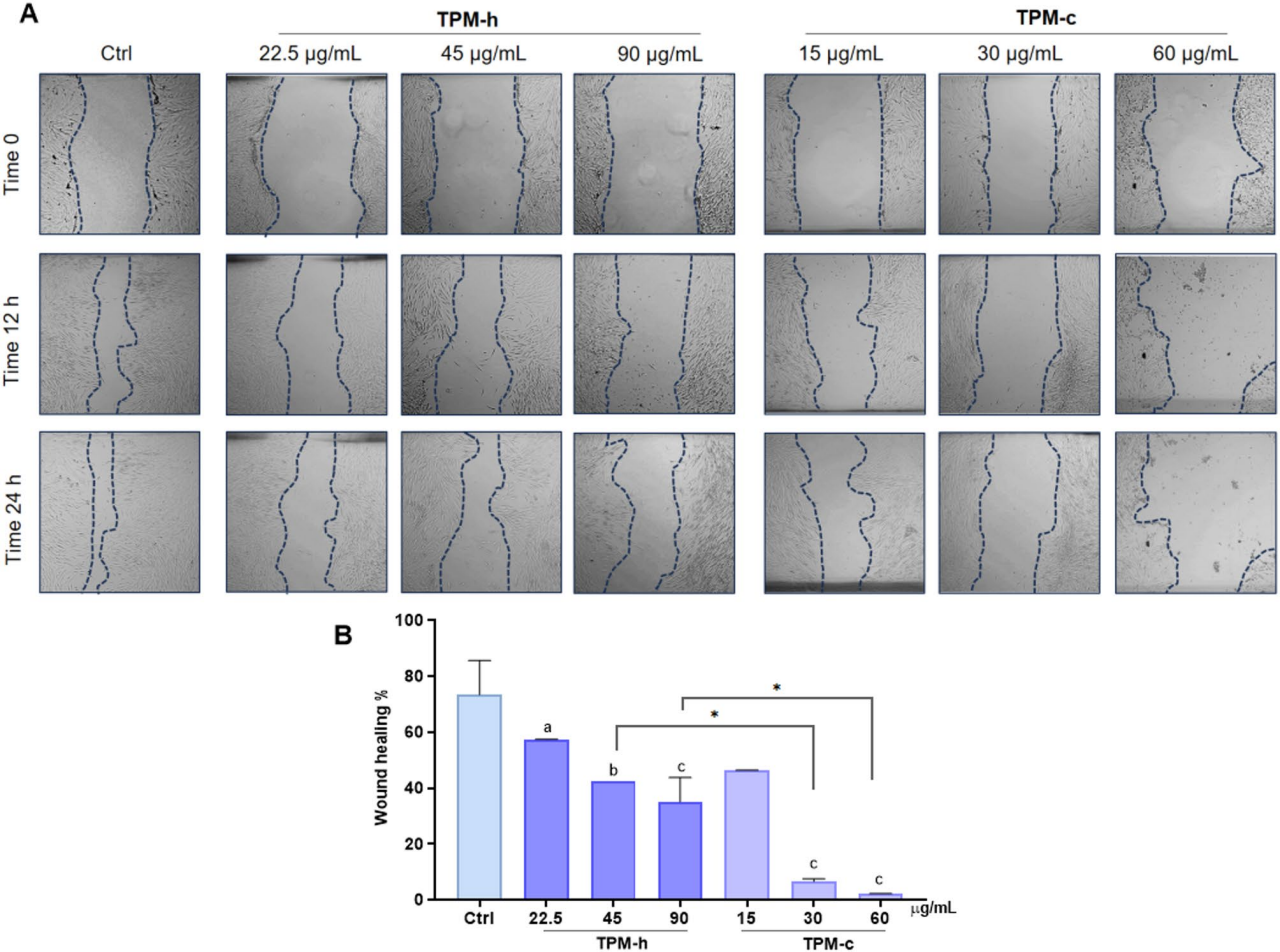
Delay in wound healing capacity of hGFs exposed to TPM-h and TPM-c. **A:** Relative wound healing images of hGF cells after exposure to TPM-h and TPM-c. **B: **Wound healing ratio in hGF cells exposed to TPMs for 24 h. Ctrl: Control group without any treatment; TPM-c: Total particulate matter derived from a conventional cigarette; TPM-h: Total particulate matter derived from a heated tobacco product. Statistical significance between groups: Ctrl vs. groups *p*^a^ < 0.05; *p*^b^ < 0.0005; *p*^c^ < 0.0001; TPM-h (45 µg/mL) vs. TPM-c (30 µg/mL) p* <0.0001; TPM-h (90 µg/mL) vs. TPM-c (60 µg/mL) p* < 0.0001. After creating a wound model for hGF cells, they were exposed to TPMs concentrations for 24 h and relative wound healing was evaluated. Data are expressed as mean ± SD of three independent experiments.

The images of delay in relative wound healing observed in hGF cells exposed to TPMs at 0, 12 and 24 h are given in Fig. 6A. According to the cell scratch assay results given at Fig. 6B, while a 70% wound healing rate was observed in the control group at 24 h, it was observed that the healing rates of both TPM groups were significantly delayed depending on the concentration compared to the control (*p*^*a*^ *< 0.05; p*^*b*^ *< 0.0005; p*^*c*^ *< 0.0001*). The results showed that the wound healing in the TPM-c was significantly declined compared to the control group. Although a similar delay in healing was observed following TPM-h exposure, the extent of this delay was slightly greater than that caused by TPM-c. These findings suggest that both TPM derivatives impair hGF cell migration capacity and may therefore negatively influence the wound healing process during periodontal regeneration in vitro.

## Discussion

Cigarette smoking has long been recognized as a major risk factor for the development and progression of periodontal diseases. Numerous epidemiological and clinical studies have demonstrated that smokers are at a notably higher risk of periodontitis, with increased clinical attachment loss, alveolar bone resorption, and poorer treatment outcomes compared to non-smokers^20,21^. This elevated risk is attributed not only to the presence of nicotine but also to the complex mixture of toxicants present in CS, which contribute to impaired host response, altered microbial colonization, and enhanced tissue destruction^12,22,23^. On the other hand, clinical trials are often limited by high costs and the longer process of recruiting patients who meet the strict inclusion criteria, whereas predictive studies, such as new approach methodologies offer a more affordable, reproducible, and efficient approach to disease modeling, making them a valuable alternative. The majority of clinical and experimental studies in the literature have been conducted with traditional cigarettes and e-cigarettes, particularly via the gas phase of mainstream smoke^16,24–30^. However, the particular phase of these products that adheres to and accumulates on gingival tissues remains largely understudied, despite its potential relevance for localized toxicity and periodontal disease progression. Recent studies have primarily focused on the overall smoke phase of both tobacco products, with particular emphasis on comparisons of aerosol emission profiles^31,32^. Moreover, limited reports have indicated that individuals using these products exhibit periodontal tissue damage mediated by inflammatory responses and oxidative stress, accompanied by alterations in the expression of key growth factors, including transforming growth factor (TGF)-β1, platelet-derived growth factor (PDGF)-AB, and VEGF^26,33–35^. Hence, we compared the chemical spectrum and toxicity impact of TPM phases of HTPs and 1R6F cigarettes in primary hGF cells to enlighten the mechanistic aspect of these products on periodontal toxicity in this study. The chemical analysis results showed that the manually extracted TPMs per product exhibited a notable difference in potential differences in product design variability, as well as their toxicity impacts. The detected level of nicotine in TPM-c was recorded as 759.43 ± 2.80 µg/mL in the present study, whereas in our another unpublished study, nicotine level was detected as 498.15 ± 3.19 µg/mL in the TPM-h fraction^36^. While previous studies have mainly focused on the TPM phase of conventional cigarettes or the mainstream smoke of both products^37–40^, demonstrated higher nicotine levels in CS extract (CSE) (38.2 mg/mL) compared to HTP-derived extracts (21 mg/mL). According to a recent comparative evaluation on the particulate emissions of conventional cigarette and HTPs indicated that HTPs significantly emitted fewer particles compared to cigarettes, with comparatively larger particles in the form of droplets^41^. However, no analysis has yet addressed the TPM phase of HTPs using organic solvent-based extraction. Accordingly, the compositional and in vitro toxicity data presented here provide a novel contribution to the literature. Furthermore, TPM-c was found to contain markedly pronounced levels of Cd, Li, and Zn compared to TPM-h according to our findings. In a previous study, relatively higher Cd concentration detected in CS (3.54 ± 0.94 µg/g) is of concern, as chronic exposure to Cd has been reported to induce dysplastic alterations in gingival tissues and up-regulate inflammatory markers such as inducible nitric oxide synthase. Hence, chronic Cd exposure may directly influence intracellular gene expression, thereby modulating inflammatory signaling pathways^42^. Furthermore, detailed nicotine and TSNAs analysis emphasized that one cigarette contains significantly higher levels of these chemicals compared to the one heatstick of HTP. Moreover, polycyclic aromatic hydrocarbons (PAH), benzo(a)pyrene, CO, aromatic amines, hydrogen cyanide, phenol, volatile organic compound (VOC, 1,3-butadiene, isoprene, acrylonitrile, benzene, toluene) levels were also remarkably higher in CSE compared to HTP aerosol. Our findings align with previous report detecting lower levels of Iron (Fe), Pb, Chromium (Cr), Aluminum (Al), As, and Nickel (Ni) in HTP aerosols exposed to animals with no detectable levels of Cd and Manganese (Mn)^43^. However, it was noted that menthol-flavored HTPs contain certain harmful constituents at levels even higher than those detected in conventional cigarettes as well^31^.

Recent data on the onset or progression of periodontal diseases associated with HTP exposure remain limited^32,40,44–50^. Our analyses on the cytotoxicity revealed that exposure to TPMs, at equivalent nicotine concentration, reduced the hGF cell viability in a dose-dependent manner. However, this effect was not observed consistently in the case of TPM-h exposure prominently. Although some studies suggested that HTPs may be less harmful than conventional cigarettes, detailed investigations regarding chronic exposure remain limited. In a recent study comparing the smoke phases of both tobacco products, HTP exposure did not elicit a dose-dependent cytotoxic response in gingival fibroblasts and even stimulated proliferation at low doses^40^. Therefore, the observation of distinct cellular effects despite equivalent nicotine exposure suggests that non-nicotine constituents play a critical role in determining inflammatory and/or regenerative death pathways. The present study demonstrated that TPM-c exerted markedly stronger cytotoxic effects on hGFs compared to TPM-h. Although both products elicited biological stress responses, the cytotoxicity potencies differed substantially, which can be explained by their distinct physicochemical profiles. First, the higher cytotoxic potential of TPM-c is consistent with the well-established chemical burden associated with combustion. Conventional CS contains significantly elevated levels of PAHs, ROS, and TPM, all of which contribute to oxidative stress, lipid peroxidation, and mitochondrial dysfunction. CS is known to generate free radicals per puff, many of which are stable long enough to interact with cellular macromolecules. In contrast, HTP systems operate at substantially lower temperatures, thereby reducing the formation of combustion-dependent toxicants^32^. Due to this chemical disparity, the more severe cytotoxic and morphological alterations might be observed in TPM-c–exposed gingival cells. Moreover, the abundance and chemical reactivity of the TPMs underline the characteristic differences between toxicity profiles. According to the previous study, TPM-c potentiated platelet reactivity to thrombin and thus increased aggregation at a concentration of 25 ~ 100 µg/mL^51^. Another report on hGFs, moreover, revealed that TPM-c led to pronounced loss of viability and enhanced collagen degradation^52^. Similar to our findings, cannabis smoke condensate induced a similar loss of viability in human gingival epithelial cells through apoptosis, elevated ROS production, and autophagy^53^. In our study, similar mechanisms may have contributed to the following TPM-c exposure. Conversely, HTP aerosols contain fewer and less chemically reactive particles and heavy metals, which may explain TPM-h’s comparatively attenuated cytotoxic effects. This finding underscores the necessity of evaluating the safety of HTPs using multiparametric approaches beyond nicotine content. Further analyses to elucidate the underlying periodontal toxicity pathways, oxidative, inflammatory, and regenerative responses were evaluated in hGF cells exposed to both fractions. Previous study revealed that TPM-c exhibited inhibitory effect on the growth of hGFs compared to the control while the effect was not dose-dependent^52^. On the other hand, recent data on the HTPs mainly focused on smoke/ aerosol phase, stated that these products have consistently demonstrated measurable cytotoxic response, albeit generally lower than that of conventional cigarettes, indicating that HTP aerosols are not biologically inert and can still elicit adverse cellular responses^6^. Similarly, two studies reported that TPM-h exposure had 20-fold less effect on mitochondrial function and viability in human bronchial epithelial cells compared to TPM-c, with markers of cellular adaptation up to 12-week exposure^54^. In addition to these results, both conventional cigarette-derived and Swedish snus-derived TPM phases were assessed in human organotypic gingival cultures for up to 72 h, where impaired biological activity and morphological deterioration were observed, particularly in group exposed to TPM-c^55^. According to our findings, despite reduced cell viability, intracellular GSH levels remained unchanged, suggesting that cell death may occur through oxidative stress–independent mechanisms such as mitochondrial dysfunction, lysosomal damage, or endoplasmic reticulum stress. In certain contexts, hGF cells may upregulate GSH synthesis in response to stress, masking depletion despite ongoing cell death^56^. Flow cytometry analyses further revealed that TPM-c exposure primarily induced necrosis, whereas TPM-h exposure under equivalent nicotine concentrations led predominantly to apoptosis. Consistent with our results, Scharf et al. (2021) reported increased rates of both early and late apoptosis as well as necrosis in Jurkat T cells following exposure to CSE and HTP, with the effects being more pronounced under CSE exposure^43^. Moreover, flow cytometry data suggested that TPM-c disrupted the intracellular homeostasis and induced direct cell death compared to TPM-h. Previous data in hGFs and keratinocytes exposed to HTP extract showed increased p53 expression and decreased Bcl2 and p21 expression in the fibroblasts, whereas in the keratinocytes, increased Bcl2 expression and decreased p53 expression was reported. In addition, p53 expression increased significantly in hGFs dose-dependently, while p21 and Bcl2 expressions were down-regulated in hGFs^57^. Apoptotic and necrotic cell death in hGFs has also been reported following exposure to both e-cigarette aerosol and conventional cigarette smoke; however, the difference in the proportion of necrotic cell death between the two groups remained insignificant up to 24 h. Moreover, caspase-3/7 activation did not show a significant difference between hGFs exposed to e-cigarette vapor and those exposed to conventional cigarette smoke^58^. Similar to the previous studies, CSE exposed-human oral mucosal epithelial cells showed increased late apoptotic response, which was reduced via rapamycin pre-treatment^59^. On the other hand, MDA, a biomarker of lipid peroxidation, was markedly elevated at 22.5 µg/mL of TPM-h, suggesting that TPM-h–induced gingival cytotoxicity may initially be driven by oxidative stress. Interestingly, MDA levels declined at higher doses (45–60 µg/mL), which may indicate either cellular adaptation through activation of antioxidant defense mechanisms or autophagosome formation. In line with these findings, MDA was detected lower with TPM-c at the tested concentrations, likely due to excessive necrosis leading to MDA leakage into the culture medium, and hence not being detected in the hGF pellets. Previous in vitro data reported that cannabis exposure led to deleterious oxidative response in human gingiva epithelial cells, via increased total ROS production^53^. Furthermore, nicotine exposure elevated total ROS levels and led to an upregulation of the antioxidant transcription factor Nrf2 in hGFs^60^. In another study, CSE exposure dose-dependently led to a significant increase in mRNA expressions of Nrf2 and hemeoxygenase-1 (HO-1) as well as total ROS production^61^. hGFs exposed to cigarette puff-stimulated PBS (0.5–12 puffs) for 1 min, exhibited a dose-dependent increase in the intracellular ROS production^9^. A recent finding on e-cig and cigarette vapors suggested that the total H_2_O_2_ production significantly higher in hGFs exposed to conventional cigarette vapor in a time-dependent manner^58^. Moreover, a comparative study in mouse endothelial cells revealed that cigarette derived-TPM (10–100 µg/mL) exhibited more potent increase in total superoxide level compared to CSE prepared by the same number of cigarettes^51^. In line with the reported data, limited findings are present in the literature based on the oxidative response induced via tobacco-product induced TPMs. Hence, based on the research hypothesis, our findings were limited to the primary markers of oxidative stress such as GSH and MDA levels and needs to be assessed more extensively in future studies. On the other hand, LC3β-II expression was up-regulated in hGFs exposed to TPM-h doses (22.5–45 µg/mL), pointing to the induction of autophagic responses aimed at counterbalancing oxidative stress. These findings are consistent with reports that autophagy activation at stress-inducing doses functions as an adaptive mechanism to promote cell survival^62,63^. Similar to our finding, CSE, representing the smoke compartment, has also been shown to induce autophagy response in gingival fibroblasts via LC3β-II up-regulation^64^. In another report, it was concluded that CSE exposure to human periodontal ligament cells (hPDLCs) led to an increase in LC3β-II expression as well as p62 accumulation. Moreover, Beclin-1 protein levels were declined in CSE exposed group, suggesting that CSE negatively interferes with osteogenic potential of hPDLCs^65^. A study in mouse oral mucosal epithelium, reported a similar findings that CS exposure significantly increased protein expression of LC3β-II and Beclin-1, whereas down-regulated p62 expression^59^. Since the present study is the first preliminary comparative data with TPMs of cigarette and HTPs, LC3β-II/I was the only marker to determine autophagy related cellular response and autophagosome formation. However, to elucidate the exact mechanism of TPM-activated autophagy, other pathways involved in autophagy such as p62, Beclin-1 and Atg5 expressions need to be addressed in further studies. In addition, the role of autophagy in inflammatory processes has been highlighted in the literature, where it has been associated with increased pro-inflammatory responses and IL-6 secretion in conditions such as periodontitis^66^. Our findings revealed that IL-6 levels were found to increase more prominently following TPM-h exposure, suggesting stronger activation of immune mechanisms and inflammatory pathways by this fraction. These findings align with previous reports indicating that HTPs and conventional cigarettes elicit distinct cytokine response profiles^67^. Moreover, IL-6 is a key mediator of the acute-phase response and inflammation, with dual roles in wound healing. Previous study by Tatsumi and colleagues reported elevated levels of IL-6 and IL-8 in hGFs either exposed to nicotine or commercially available TPM-c up to 25 days^68^. Our results indicated that TPMs may elicit low-level inflammatory responses; however, the overall low levels of inflammation observed suggest that exposure at these concentrations may not trigger strong inflammatory activation. Future studies should include additional inflammatory markers (e.g., TNF-α, IL-1β) to comprehensively assess the immunomodulatory effects of TPM fractions in gingival tissue. Interestingly, despite its pronounced cytotoxic effects on cell viability, TPM-c slightly induced pro-inflammatory IL-6 and regenerative VEGF-A responses. This discrepancy may be linked to its higher heavy metal content or other volatile toxic constituents, which may directly suppress intracellular signaling pathways or induce mitochondrial damage, thereby promoting cell death without stimulating inflammatory signaling. Clinical findings on inflammatory markers mainly focused on smoking or vaping correlation, and one of the reports highlighted that cigarette smokers exhibited increased periodontal inflammation in line with increased plaque index, bleeding on probing and probing depth compared to non-smokers and e-cig users^69^. Another clinical follow-up reported that e-cig smokers exhibited higher gingival inflammation compared to non-smokers in correlation with the change in saliva components^70^. On the other hand, a clinical study reported decreased gingival inflammation in heavy smokers compared to light and medium smokers, which was attributed to the masking effect of heavy smoking^71^. In another clinical report among e-cig, Swedish snus and nicotine pouches users, it was indicated that Swedish snus users exhibited a remarkable increase in saliva IL-1, IL-6, IL-8, and TNF-α levels compared to the other groups, which was follwed by e-cig users^72^. Hence, it might be suggested that both experimental and clinical studies on inflammation and smoking overall represents findings with smoke extracts or real-life smoking conditions. In addition, MMP-9, which plays a crucial role in extracellular matrix remodeling and wound healing, showed no significant changes in response to TPM-h or TPM-c exposure. This suggests that the tested agents did not exert sufficient proteolytic activity to affect extracellular matrix dynamics under the conditions. However, given that MMP-9 is typically elevated during later phases of inflammation rather than at early stages, the 24 h exposure may have been insufficient to detect significant induction. Early activation of other metalloproteinases, such as MMP-1 and MMP-2, has been documented in wound healing^40^. Hence, further studies should therefore examine multiple MMP isoforms (MMP-1, -2, -3, -8) and tissue inhibitors of metalloproteinases (TIMPs) to provide a more comprehensive understanding of the effects on the cellular microenvironment. According to a clinical finding, cigarette smokers exhibited higher levels of MMP-8 and MMP-9 levels in their saliva samples compred to non-smokers and e-cig users^73^. Furthermore, wound-healing assays further revealed that both TPMs impaired gingival fibroblast migration, the most notably, TPM-c exhibited a stronger inhibitory effect on the regenerative capacity of gingiva fibroblasts. Since cell migration is critical during the initial stages of wound healing, this inhibitory effect could lead to substantial delays in periodontal tissue regeneration. Similar reports in the literature indicate that smoke-phase extracts of both conventional cigarettes and HTPs negatively affect gingival fibroblast migration and proliferation^40^. A study on tissue regeneration of hGFs in vitro revelaled that TPM-c exposure remarkably increased MMP-2, MMP-14, TIMPs, and decreased collagen degredation, whereas nicotine exposure has slight effect on these parameters^74^. Our findings extend these observations to TPM fractions, showing that TPM-c markedly impaired migration capacity, while TPM-h was comparatively less detrimental. The inhibitory effects observed are consistent with previous evidence demonstrating that nicotine reduces proliferation and adhesion of periodontal cells^23^. Nonetheless, the divergent proliferative capacities observed between the two fractions under equal nicotine exposure point to the role of other constituents, such as heavy metals or volatile combustion products. A more detailed mechanistic evaluation will be crucial to better understand acute and chronic damage–regeneration dynamics. Collectively, these results support the notion that TPMs possess distinct chemical compositions, leading to divergent cytotoxic and stress-inducing profiles.

The present findings highlight the greater potency of TPM-c in inducing cytotoxicity, oxidative stress, inflammation, and impairing tissue remodeling in hGF cells compared to TPM-h. In agreement with these concerns, our findings indicate that while the TPM-h fraction exhibited relatively lower cytotoxicity compared to TPM-c, it nevertheless induced pro-inflammatory and angiogenic responses. These results highlight that HTPs are not devoid of toxicity and can trigger cellular inflammatory responses in gingival tissues. It’s interesting to note that oxidative stress and inflammatory marker profiles showed complex, concentration-dependent effects. TPM-c, possibly as a compensatory response to cellular stress, induced an increased angiogenic response through VEGF-A. These different reactions demonstrate how exposure concentration and composition of chemicals affect tobacco-related cytotoxicity. To clarify the long-term implications of tobacco exposure on oral health and to inform harm-reduction strategies in clinical practice, continued research employing predictive models is essential.

## Methods

### Preparation of TPM

The extraction of TPM was carried out according to our previous studies^7,8^. Briefly, one of the 1R6F research cigarettes (University of Kentucky, Kentucky, USA) and a heat stick (using its heater device) were burned/heated separately according to the ISO-Health Canada Intense (HCI) Puff Regimen Protocol^75^. For TPM collection on the Whatman filter papers were first weighed and recorded as “Weight of blank Whatman paper.” The blank was then placed inside the extraction filter unit, and the TPM generated during the cigarette/HTP combustion process was deposited on the blank Whatman paper. After the extraction process, the filter was weighed again to determine the accumulated particulate mass. The accumulated TPM was then calculated based on the weight change of the filter paper in the collection unit after the extraction. This process was repeated using two more filters, and the average of the three calculated masses yielded TPM values ​​for each extracts (TPM-c and TPM-h). TPM isolated from traditional cigarettes was named TPM-c; TPM isolated from HTP was named TPM-h. At the end of the extraction, TPM-c (24 mg/mL) and TPM-h (36 mg/mL) were collected and incubated in DMSO with shaking for 24 h at 37 °C based on the reported method^51^. The prepared TPMs were stored at -80 °C for chemical analysis and in vitro studies up to one month. Moreover, the extraction scheme used in the experiment is shown in Supplementary Fig. S5.

### Chemical analysis

#### Nicotine concentration

The nicotine levels of the prepared TPMs were determined by the modification of previous study^8^. In this context, the nicotine levels in TPM extracts were analyzed using a Chromolith High Resolution RP18e monolithic column (100 × 4.6 mm, Merck, Darmstadt, Germany). The chromatographic conditions were as follows: mobile phase, 10 mM phosphate buffer (pH 7) containing 0.3% triethylamine: acetonitrile (87.5:12.5, v/v); flow rate, 1 mL/min; column oven temperature, 25 °C; detection wavelength, 260 nm; and injection volume, 10 µL. The obtained extracts were diluted at a ratio of 1:10 and directly injected into the system. Each measurement was conducted in triplicate using independently prepared TPM extracts, and the results were expressed in µg/mL. Pure DMSO was used as a blank control.

#### Heavy metal quantification

The quantitative analysis of heavy metals As, Cd, Cobalt (Co), Cr, Mn, Pb, Tin (Sn), Selenium (Se), and Zn in TPMs was analyzed by ICP-MS (iCAP Q, Thermo Scientific, USA). Briefly, TPMs and the solvent DMSO were examined in three independent experiments. Results were expressed as mean ± SD in µg/L. Sample digestion was carried out with 2 mL of ultrapure 65% (m/m) HNO_3_ and 0.5 mL of 30% (v/v) H_2_O_2_ at 80 °C for 2 h. After digestion, ultrapure water (Milli-Q system, resistivity > 18.2 MΩ·cm at 25 °C) was added to adjust the final volume to 10 mL. For ICP-MS analysis, sample solutions were further diluted 1:10 with 2% HNO_3_ solution containing 500 µg/L Au and 10 µg/L internal standards (IS) (Bismuth (Bi), Gallium (Ga), Indium (In), Iridium (Ir), Li, Rhodium (Rh), Scandium (Sc), Yttrium (Y). Procedure blanks were obtained from blank filters digested under the same conditions, and their average concentration was subtracted from the TPM-c and TPM-h values. The calculation of heavy metal content in TPM (µg/g) was performed according to Supplementary Formula S1. The LoD for each element was estimated by the formula according to the standard deviation of the blank samples with Supplementary Formula S2.

### Cell culture studies

#### Isolation, generation, and characterization of hGFs

hGFs constitute the major component of gingival connective tissue, and gingival health relies on their essential roles in repair and regeneration. Clinical studies are often limited due to high costs and the difficulty of recruiting eligible patients. However, predictive studies are cost-effective and reproducible, thus serving as a preferred alternative to clinical studies. All experimental procedures involving human gingival tissue samples were conducted in accordance with the principles of the Declaration of Helsinki and in compliance with all relevant institutional guidelines and regulations. Gingival tissue samples were obtained from two healthy adult male volunteers during routine dental procedures. Written informed consent was obtained from all participants prior to sample collection. An Ethics Committee Approval (No: 2025-02/90) was received from Acibadem Mehmet Ali Aydinlar University for the preparation of human gingival tissue samples used for predictive studies in the study. Human gingival tissue was obtained from a healthy, non-smoking adult without chronic disease or medication use. The tissue was collected in sterile 0.9% sodium chloride (NaCl) on ice and immediately processed. Samples were cut into ~ 1 cm^2^ pieces under sterile conditions and washed three times with cold Dulbecco’s phosphate-buffered saline (DPBS, w/ Ca^2+^, Mg^2+^, Gibco, New York, USA) supplemented with 1% penicillin-streptomycin (Gibco, New York, USA). Tissue fragments were digested using collagenase type-I (Gibco, New York, USA) at 37 °C to release cells, which were then resuspended in high-glucose DMEM containing 10% FBS (Gibco, New York, USA), 100 U/mL penicillin, and 100 µg/mL streptomycin. The suspension was centrifuged (300 g, 5 min), the pellet was resuspended, and cells were seeded into T-25 flasks (Isolab, Wertheim, Germany). Cultures were maintained at 37 °C in a 5% CO₂ incubator, with medium changed every 2–3 days and passaging every 5–7 days via trypsinization. Early-passage cells were expanded, cryopreserved, and used for subsequent experiments.

**Fig. 7:**
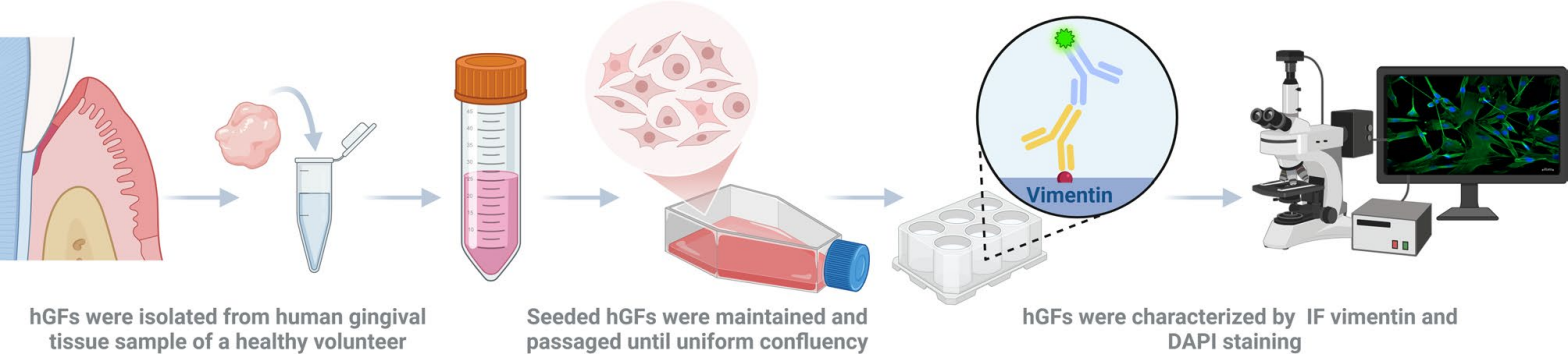
Schematic diagram for hGF cell isolation and characterization. DAPI: 4′,6-diamidino-2-phenylindole; hGF: Human gingival fibroblasts; IF: Immunofluorescence. (Created in BioRender^36^. https://BioRender.com/zphoz31)

For the characterization of isolated cells, seeded cells were fixed with 4% paraformaldehyde (PFA, Massachusetts, USA) for 20 min at room temperature. Followingly, the cells were washed with cold-DPBS three times for 5 min and permeabilized with 0.1% Triton X-100 (Dow Chemical, #A9780, Michigan, USA) for 15 min at room temperature. After following a similar washing procedure, each well plate was blocked with 3% bovine serum albumine (BSA, Sigma Aldrich, #05470, Darmstadt, Germany) for 40 min at room temperature and incubated overnight at 4 °C in anti-vimentin primary antibody solution (Vimentin Polyclonal Antibody, Elabscience, # E-AB-33217, Texas, USA) diluted 1:200 or 1:500 with BSA (1.5%, Sigma Aldrich, Darmstadt, Germany). The following day, the cells were washed 3 times with DPBS for 5 min and labeled with fluorescein isothiocyanate (FITC)-labeled secondary antibody (CoraLite488-conjugated Goat Anti-Rabbit IgG(H + L), Uden, Netherlands) diluted 1:600 ​​with BSA (1.5% w/v) and incubated for 1 h at 37 °C. At the end of incubation, cells are kept at room temperature for 10 min with DAPI for nuclear staining. Then, hGF cells were examined under a fluorescence microscope (Olympus, IX53, Tokyo, Japan) at 5x, 20x, and 40x magnification for further imaging^76^ (Fig. 7).

#### Cytotoxicity assay

The cytotoxicity profiles of TPMs in hGF cells for 24 h were evaluated spectrophotometrically with the MTT test according to the described study^8^. Each experiment was performed independently in triplicate, and the results were expressed as relative cell viability compared to the negative control. IC_50_ values of TPM samples were calculated according to the effective concentration interpolation calculation formula via Microsoft Excel, based on the dose–response data as mentioned previously^77^.

#### TPMs mediated apoptotic and necrotic cell death

In order to assess apoptotic/ necrotic cell death induced by TPMs, the Annexin V-FITC staining kit (Annexin V-FITC Kit, Elabsicence, #E-CK-A211, Texas, USA) was used for the quantitative detection of apoptotic and necrotic cell death according to a previously described method in detail^7^. Briefly, cells (10^6^ cells/mL) were seeded in 12-well plates and treated with TPMs for 24 h at doses below and above the determined IC_50_ values. 5% DMSO (v/v) was used as a PC for apoptotic cell death, whereas cells treated with medium were evaluated as the negative control.

#### Autophagosome formation

Autophagy is a natural and regulated physiological mechanism underlying the degradation of damaged organelles and recycling of cytoplasmic materials involved in multiple pathophysiologies, and recent studies report that this mechanism is induced by inflammatory damage in periodontal tissue^78^. Briefly, hGF cells cultured in confluent T-25 flasks were exposed to both TPMs at concentrations below and above of IC_50_ value for 24 h. To detect the protein expression of microtubule-associated protein 1 A/1 B-light chain 3-phosphatidyl ethanolamine conjugate/LC3-II (LC3β, Elabsicence, Texas, USA), 20 µg protein was loaded for each group based on the previous protocol given in detail^7^. Full-length, uncropped Western blot images and membrane images were also included in Supplementary Figure S6-S9.

#### Oxidative and inflammatory response

Oxidative stress mediated by TPMs in hGFs was evaluated through the detction of intracellular GSH level and, MDA level, as a lipid peroxidation biomarker. Triplicate measurements of GSH levels were expressed as µmol/g protein, whereas MDA levels were expressed as nmol/g protein as described previously^8,19^. VEGF-A and IL-6 levels were quantified in hGF cell supernatants following TPMs exposure using a human VEGF-A ELISA kit (Elabscience, #E-EL-H0111, Texas, USA) and Human IL-6 ELISA kit (Elabscience, E-EL-H6156, Texas, USA). Measurements were performed according to the manufacturer’s instructions, and results were expressed in pg/mL (*n = 2*).

#### In vitro *cell migration*

The cell scratch assay was performed to evaluate the impact of TPMs on the delay of gingival wound healing in vitro. A vertical scratch was created using a 0.2 mL pipette tip, after which TPMs were applied at concentrations determined by MTT assay results (*n* = 3). Images were captured at 0, 12, and 24 h post-exposure using a Zeiss Primovert Light Microscope (10×, Baden-Württemberg, Germany), and wound closure was quantified with ImageJ software (NIH, Java, Maryland, USA) according to the Supplementary Formula S3 as described previously^19^.

### Statistical analysis

Statistical analyses were performed using GraphPad Prism 9.0 (GraphPad Software, San Diego, CA, USA), as previously described^19^. For comparisons among more than two groups, normally distributed data were analyzed using one-way or two-way ANOVA followed by Tukey’s post-hoc test, whereas the Kruskal–Wallis test followed by Dunn’s multiple comparisons test was used for non-parametric data. Data are presented as mean ± SD, and statistical significance was set at *p* < 0.05.

## Supplementary Information

Below is the link to the electronic supplementary material.

Supplementary Material 1

## Data Availability

The datasets used and/or analysed during the current study are available from the corresponding author on reasonable request.
