# Supplementary material for: Tobacco-derived particulates and the periodontal axis: Distinct cytotoxic and stress-related mechanisms in human gingival fibroblasts
Source: Sci Rep. 2026 Feb 24;16:10387. doi: 10.1038/s41598-026-35317-8 (PMC13031322; doi:10.1038/s41598-026-35317-8)
Supplement: Supplementary file 1 — Supplementary Material 1 [file 41598_2026_35317_MOESM1_ESM.docx]

**Tobacco-derived particulates and the periodontal axis: Distinct cytotoxic and stress-related mechanisms in human gingival fibroblasts**

Kolci K.^1,2^, Öz E.^1,3^, Yildirim S.^4^, Azevedo R.^5^, Gungormek H. S.^6^, Almeida A. ^5^, Reis R.^1*^

^1^Acibadem Mehmet Ali Aydinlar University, Department of Toxicology, Faculty of Pharmacy, İstanbul/TURKİYE

^2^ Yeditepe University, Department of Toxicology, Institute of Health Sciences, Istanbul/TURKİYE

^3^ Istanbul University, Department of Toxicology, Institute of Health Sciences, Istanbul/TURKİYE

^4^ Karadeniz Technical University, Faculty of Pharmacy, Department of Analytical Chemistry, Trabzon/TURKİYE

^5^Network of Chemistry and Technology/Associated Laboratory for Green Chemistry (REQUIMTE/LAQV), Department of Chemical Sciences, Faculty of Pharmacy, University of Porto, Porto, Portugal

^6^ Marmara University, Department of Periodontology, Faculty of Dentistry, Istanbul/TURKİYE

*Corresponding author: [rengin.reis@acibadem.edu.tr](mailto:rengin.reis@acibadem.edu.tr)


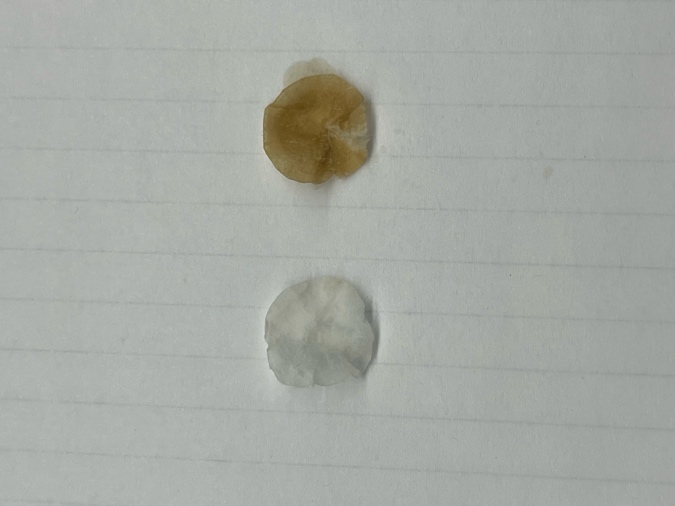


B

A

**Supplementary Figure S1**. Collected TPM-h (A) and TPM-c (B) on Whatman filter papers

TPM-c: Total particulate matter derived from a conventional cigarette; TPM-h: Total particulate matter derived from a heated tobacco product.

**Supplementary Figure S2**.Nicotine calibration curve


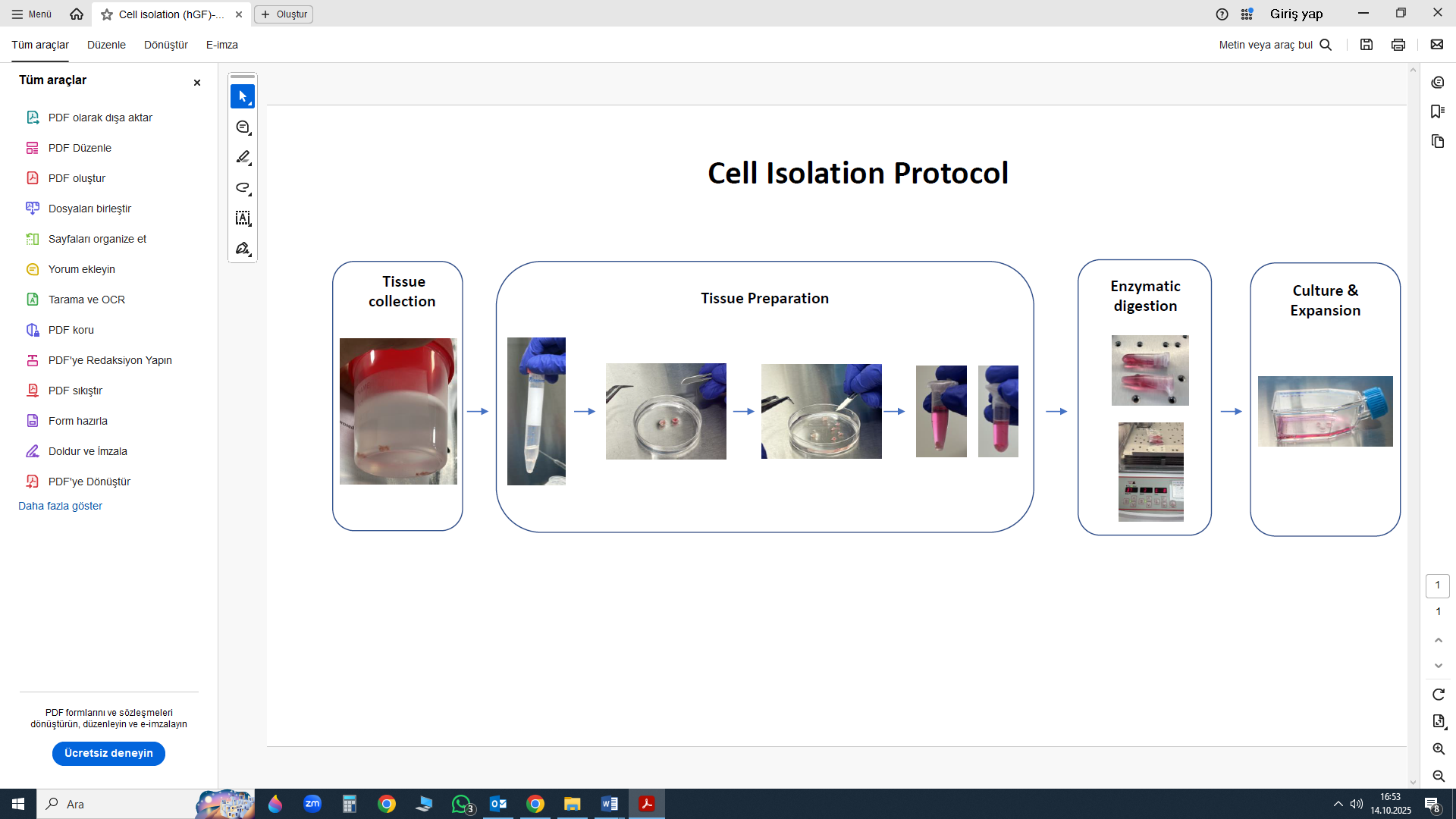


**Supplementary Figure S3**. Isolation procedure of hGFs from human gingival tissue step-by-step.


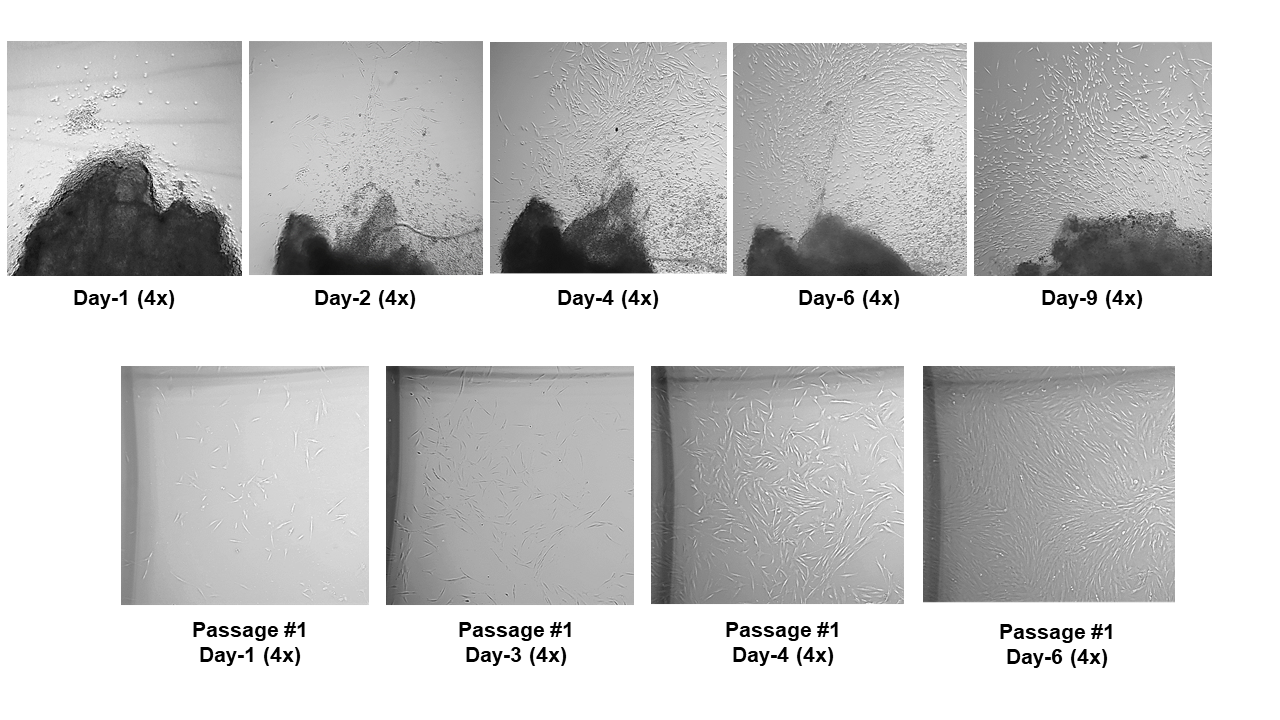


**Supplementary Figure S4**. Image of hGF cells isolated and passaged from human gingival tissue

A: Nine-day image of T-75 flask after cell isolation from tissue (4x magnification); B: Confluency of cultured hGF cells after the first passage (4x magnification). hGF: Human gingival fibroblasts


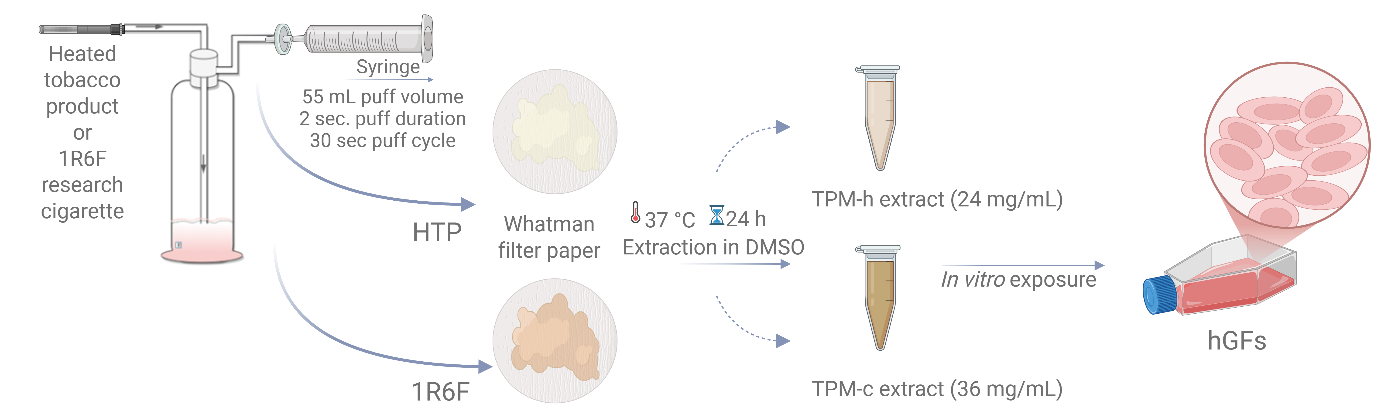


**Supplementary Figure S5**. Extraction scheme for TPM phases isolated from cigarettes and HTP (Created in BioRender. Reis, R. (2026) https://BioRender.com/im8v0k2)

TPM-h: Total particulate matter phase extraction from heated tobacco product; TPM-c: Total particulate matter phase from 1R6F research cigarette.

**Table S1.** Level of TPM accumulated on Whatman paper

|  | **Parameters (mg±SD)** | | | |  | |  | |
| --- | --- | --- | --- | --- | --- | --- | --- | --- |
| **Type of TPM** | **Weight of blank Whatman paper** | **Weight of Whatman paper after extraction** | **Weight of TPM** | **Volume of solvent** | | **Concentration of TPM** | |  |
| **TPM-h** | 19.13±1.76 | 39±3.89 | 19.87±2.2 | 827.9 µL | | 24 mg/mL | |  |
| **TPM-c** | 19.4±1.9 | 34.17±0.15 | 14.77±1.92 | 410.3 µL | | 36 mg/mL | |  |

TPM: Total particulate matter; TPM-h: TPM extraction from http; TPM-c: TPM extraction from traditional cigarettes.*n=3*

**Formula S1.**

$$TPM concentration (\mu g/g)=\frac{\frac{\left( \left[ TPM \right]-\left[ Blank \right] \right)x Vf}{1000}}{TPMweight}$$

*[TPM]: Element concentration (µg/L) in solutions obtained from acid digestion of TPM-c and TPM-h filters*

*[Blank]: Element concentration (µg/L) in solutions obtained from blank filters*

*V_f_: Final volume of digestion solution (mL)*

*TPM_weight_: Mass of TPM (g)*

**Formula S2.** LoD level calculation formulas

$$LoD levels for the elements=\frac{\frac{STDblanks x 3.3 x Vf}{1000}}{Mean (TPMweight)}$$

*STD_blanks_: Standard deviation of the blanks*

*V_f_: final volume of digested solution (10 mL)*

*Mean_TPMweight_: mean value of the mass of TPM*

**Formula S3:**

$$Wound healing \left( \% \right)= \frac{(Wound area t0) -(Wound area tfinal)}{Wound area tfinal)}x 100$$

*t_0_: The moment when the wound model was created and the first image was taken*

*t_final_: The moment when the negative control group was completely closed*

**Western Blot Raw Data**

TPM-c 30 mg/mL

TPM-h 90 mg/mL

TPM-h 22.5 mg/mL

TPM-h 45 mg/mL

TPM-c 60 mg/mL

TPM-h 90 mg/mL

TPM-h 22.5 mg/mL

TPM-c 15 mg/mL

TPM-c 30 mg/mL

TPM-h 45 mg/mL

TPM-c 60 mg/mL


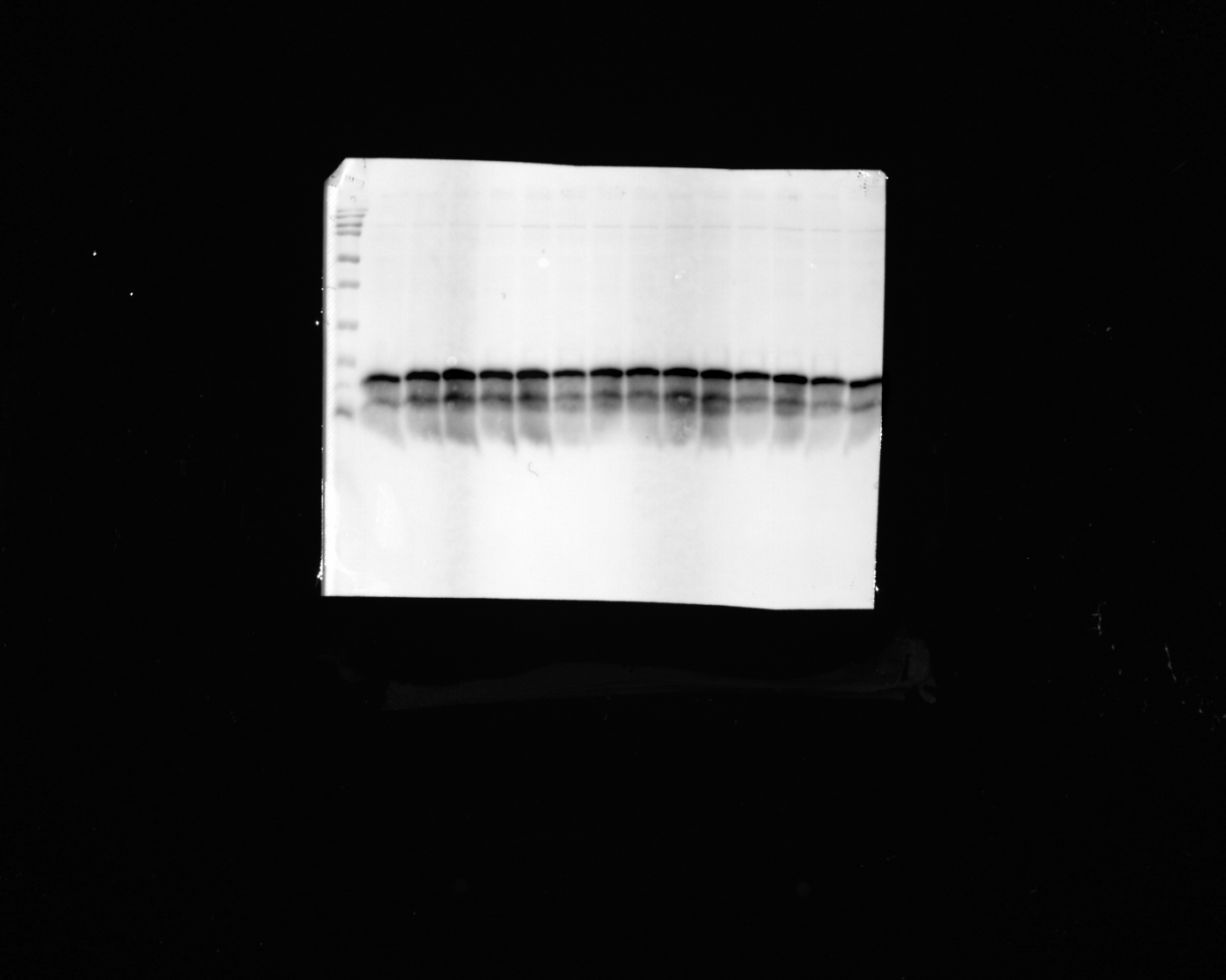


Proteins that were isolated from batch #2

Proteins that were isolated from batch #1

15 kDa

LC3β-II (16 kDa)

20 kDa

25 kDa

37 kDa

50 kDa

250 kDa

75 kDa

100 kDa

150 kDa

TPM-c 15 mg/mL

Protein ladder

Control

Control

LC3β-I (18 kDa)

**Supplementary Figure S6.** Full-length, uncropped Western blot image for LC3β corresponding to Figure 4A.

Protein ladder: Precision Plus Protein Dual Color Standards, 500 µl #1610374, BioRad.


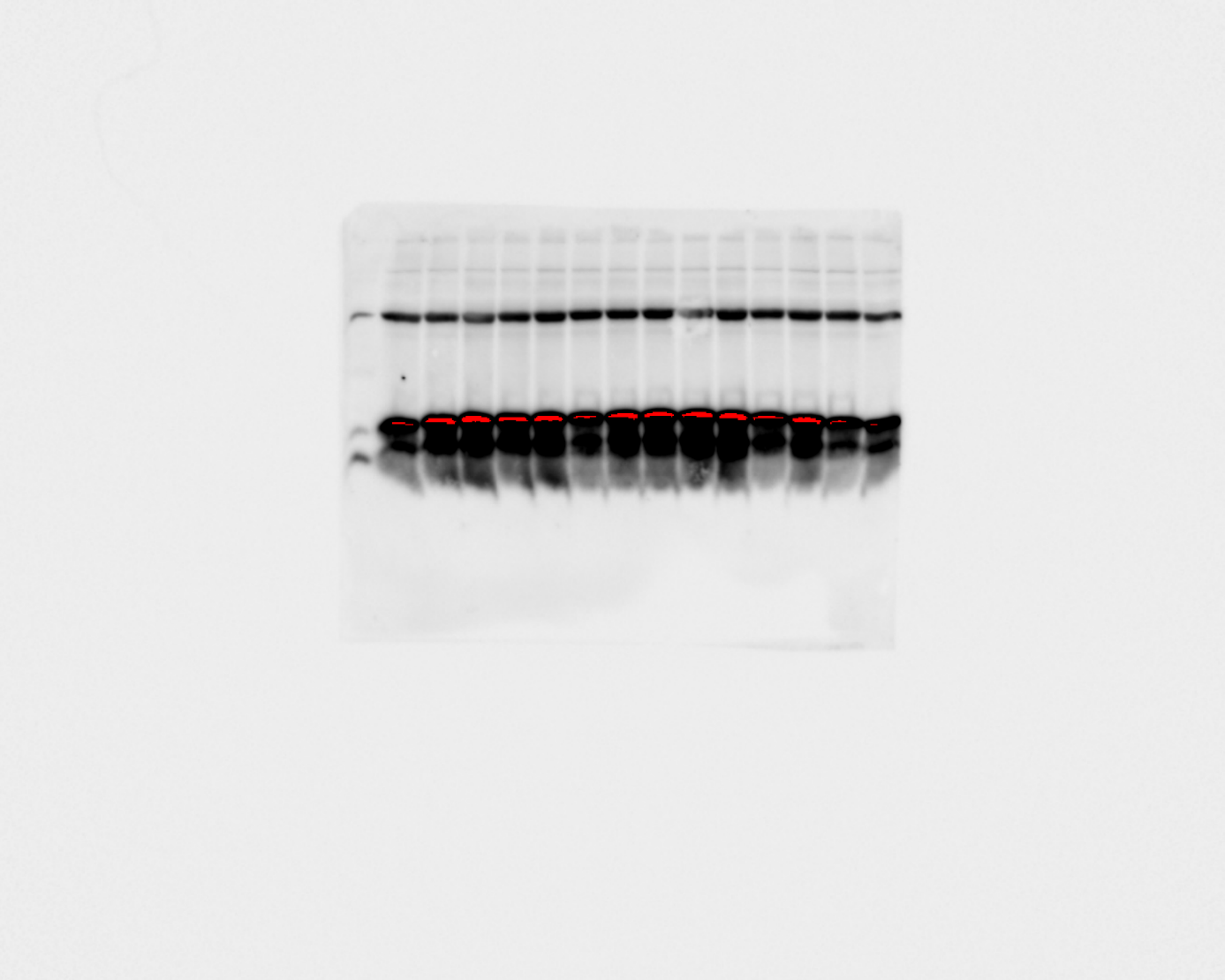


50 kDa

Proteins that were isolated from batch #2

Proteins that were isolated from batch #1

β-actin (50 kDa)

Control

TPM-h 22.5 mg/mL

TPM-h 90 mg/mL

TPM-c 30 mg/mL

TPM-h 45 mg/mL

Control

Protein ladder

TPM-c 15 mg/mL

TPM-h 22.5 mg/mL

TPM-h 45 mg/mL

TPM-c 15 mg/mL

TPM-c 60 mg/mL

TPM-h 90 mg/mL

TPM-c 60 mg/mL

TPM-c 30 mg/mL

**Supplementary Figure S7.** Full-length, uncropped Western blot image for β-actin corresponding to Figure 4A.


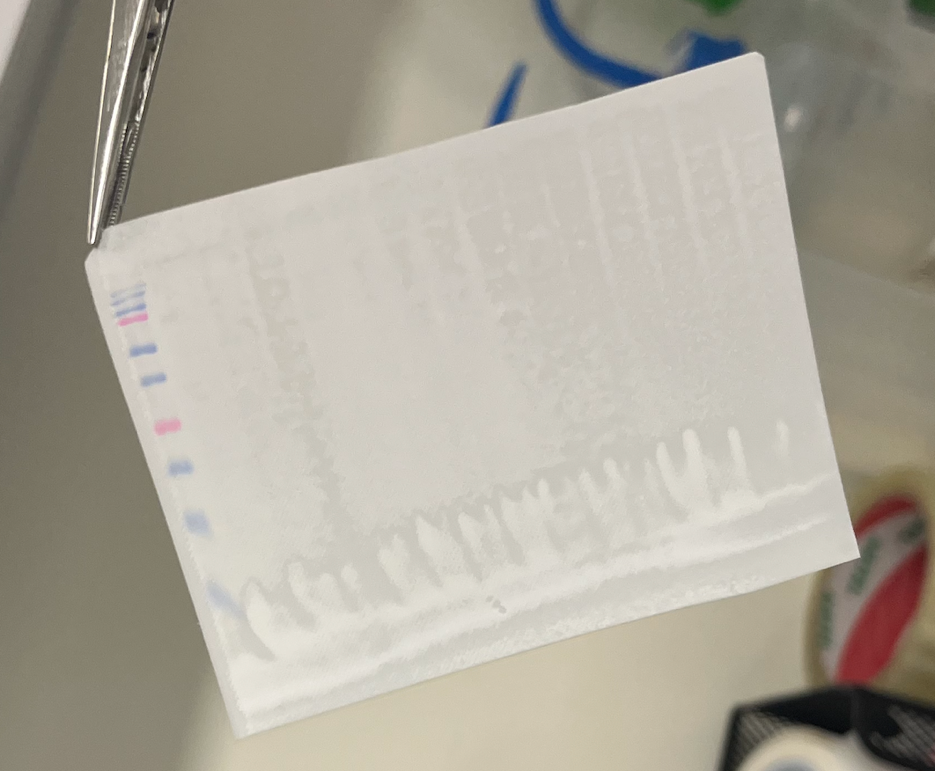


**Supplementary Figure S8.** Original image of the membrane after the wet transfer procedure


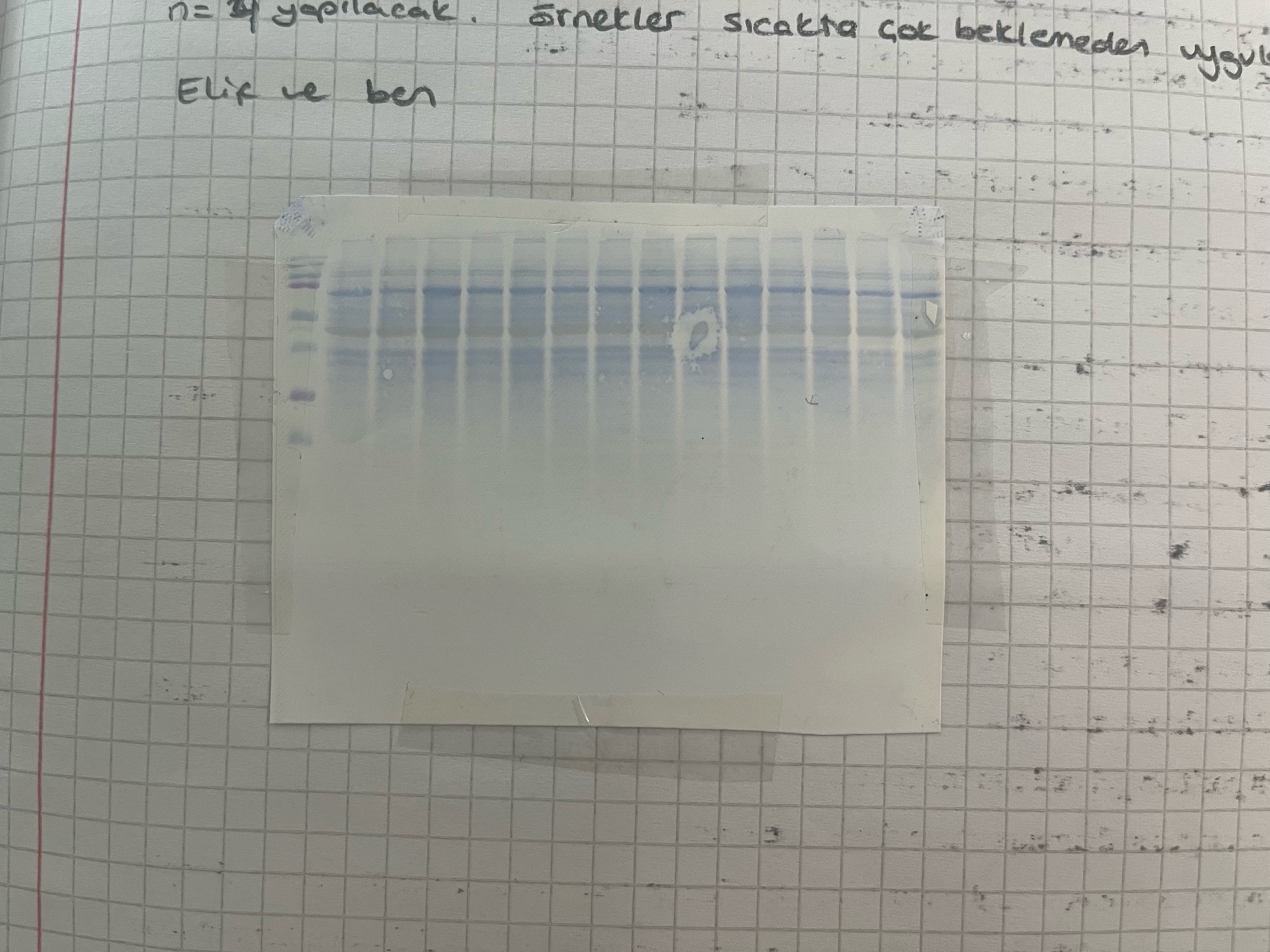


**Supplementary Figure S9.** Original image of the membrane after commasie dying
